# BEAPP: The Batch Electroencephalography Automated Processing Platform

**DOI:** 10.3389/fnins.2018.00513

**Published:** 2018-08-07

**Authors:** April R. Levin, Adriana S. Méndez Leal, Laurel J. Gabard-Durnam, Heather M. O’Leary

**Affiliations:** ^1^Department of Neurology, Boston Children’s Hospital, Boston, MA, United States; ^2^Laboratories of Cognitive Neuroscience, Division of Developmental Medicine, Boston Children’s Hospital, Harvard Medical School, Boston, MA, United States; ^3^Center for Rare Neurological Diseases, Atlanta, GA, United States

**Keywords:** EEG, electroencephalography, signal processing, batch, automated, reproducibility, MATLAB

## Abstract

Electroencephalography (EEG) offers information about brain function relevant to a variety of neurologic and neuropsychiatric disorders. EEG contains complex, high-temporal-resolution information, and computational assessment maximizes our potential to glean insight from this information. Here we present the Batch EEG Automated Processing Platform (BEAPP), an automated, flexible EEG processing platform incorporating freely available software tools for batch processing of multiple EEG files across multiple processing steps. BEAPP does not prescribe a specified EEG processing pipeline; instead, it allows users to choose from a menu of options for EEG processing, including steps to manage EEG files collected across multiple acquisition setups (e.g., for multisite studies), minimize artifact, segment continuous and/or event-related EEG, and perform basic analyses. Overall, BEAPP aims to streamline batch EEG processing, improve accessibility to computational EEG assessment, and increase reproducibility of results.

## Introduction

Electroencephalography (EEG) offers tremendous opportunities as a window into brain function. It offers potential in understanding large-scale neural network activity, and thus serves as a bridge between neurons and behavior. It is translatable, providing opportunities to bridge between animals and humans. It can be used in a variety of ages without requiring sedation, and in both typical and clinical populations, facilitating data acquisition in populations (such as young infants) who cannot follow instructions and have a limited behavioral repertoire. Its portability and relative affordability allow it to be easily used for multisite studies, which is of particular utility when studying rare diseases or acquiring large datasets.

Often the most productive research combines knowledge from two or more previously disparate fields. EEG is of particular interest to researchers in fields such as neuroscience, psychology, and development, given its ability to measure and thus enhance understanding of brain activity across a wide variety of ages, settings, and disease states. Engineers, mathematicians, and computer scientists have developed numerous signal processing techniques and tools applicable to the large amount of EEG data even a short recording session can produce. In order to bridge these fields, and thus maximize the insights that can be obtained from EEG data, brain researchers must be able to access procedures and code created by experienced signal processors.

Open source toolboxes such as EEGLAB (Delorme and Makeig, 2004), FieldTrip (Oostenveld et al., 2011) with SPM integrated (Litvak et al., 2011), Brainstorm (Tadel et al., 2011), MNE (Gramfort et al., 2014) with MNE-Python (Gramfort, 2013), and NUTMEG (Tannous and Teng, 2011) offer myriad opportunities in this regard. All of these toolboxes offer multiple advanced options for EEG (as well as magnetoencephalography, MEG) signal processing, with options to create analysis scripts for batch processing across multiple processing steps and analyses, for multiple EEG files.

Despite these important advances, accessibility of integrated analyses remains limited in some cases. For users without coding experience, creating an analysis script or pipeline may be a daunting process. For users comfortable creating such scripts, keeping track of the inputs, outputs, and specific settings in various steps of an analysis can be challenging. This is particularly a concern for users evaluating large multisite or longitudinal datasets, in which native EEG format, sampling rates, electrode layouts during acquisition, variable names, and even line noise frequencies (for international studies) may differ across files.

Additionally, reproducibility of analyses remains limited. Among automated analyses, the computer code that researchers use to generate data may link multiple software packages, set parameters that are only partially reported in a “Materials and Methods” section, and may not necessarily be saved or shared (Peng, 2011). Furthermore, “hand-editing” of EEG data further contributes to this concern, due to difficulties with human error and judgment discrepancies. Allowing reviewers or other researchers to exactly repeat prior analyses and review the details thereof would help address this concern.

The Batch EEG Automated Processing Platform (BEAPP) thus aims to aid accessibility and reproducibility by building upon preexisting EEG analysis toolboxes to provide a flexible structure for automated batch processing of EEG datasets. Beginning with raw or partially preprocessed data, BEAPP offers a series of automated steps to manage EEGs collected across multiple acquisitions setups, minimize artifact, perform several types of re-referencing, segment continuous and/or event-related EEG, and conduct basic time-frequency analyses. User inputs are determined in a single scripted user interface or in a graphical user interface (GUI) that can be saved as a template for future users, allowing users to determine their analyses and parameters without writing their own code. BEAPP tracks the output and parameters of each step of the analysis, allowing users to review prior steps or re-run a portion of the analysis with new parameters when needed.

The remainder of this manuscript provides an overview of the BEAPP format and current options, along with a series of sample analyses of publicly available data. This manuscript is intended to be used in conjunction with the BEAPP software package, user manual, and new module creation starter guide, available as described below.

## Materials and Methods

BEAPP is modular, MATLAB-based software, with user inputs entered via a GUI or a script, and with functions in script format. BEAPP is freely available, covered under the terms of the GNU General Public License (version 3) (Free Software Foundation, 2007). The BEAPP software package, user manual, and new module creation starter guide are available at: https://github.com/lcnbeapp/beapp. BEAPP is hosted on GitHub, with the intention that users adding new functionality will build upon the basic BEAPP structure, ultimately providing shared functionality for EEG analysis across a variety of laboratories and research studies. BEAPP integrates code from several other EEG analysis toolboxes and pipelines, including EEGLAB (Delorme and Makeig, 2004), the PREP pipeline (Bigdely-Shamlo et al., 2015), the CSD toolbox (Kayser and Tenke, 2006a,b), REST (Dong et al., 2017), Cleanline (Mullen, 2012), FieldTrip (Oostenveld et al., 2011), MARA (Winkler et al., 2014), and HAPPE (Gabard-Durnam et al., 2018).

**FIGURE 1:**
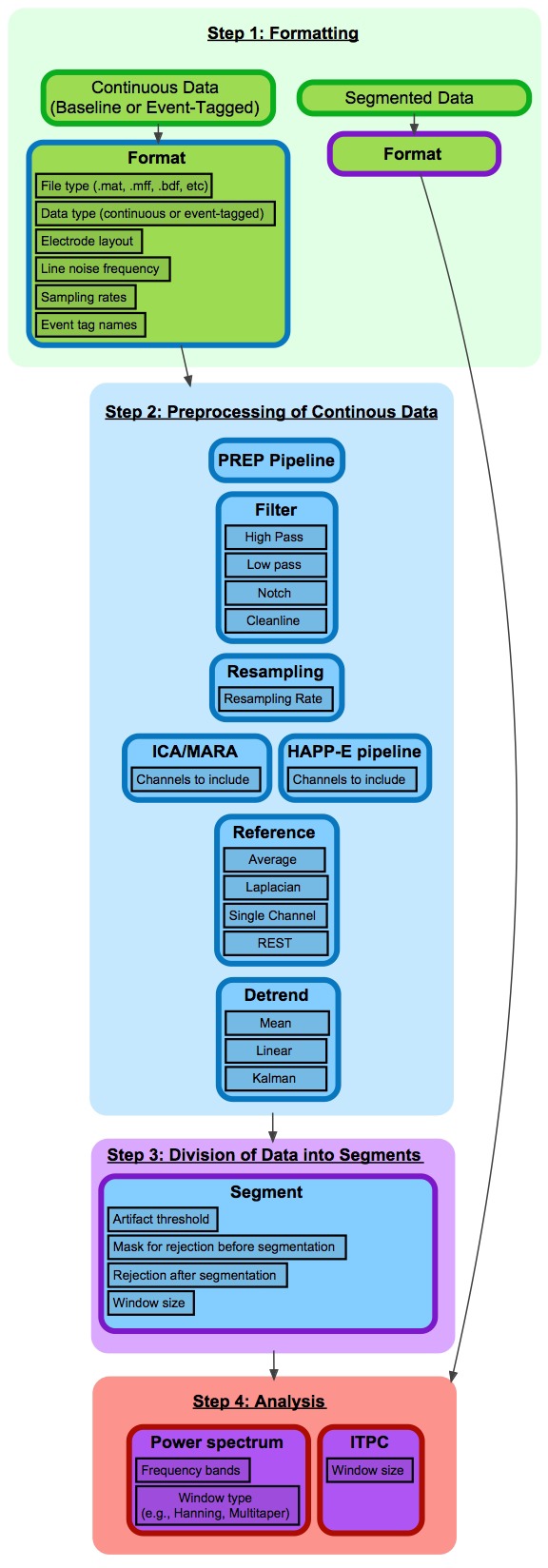
Flowchart of BEAPP steps and modules. This figure provides an overview of BEAPP. Each step is divided into modules, which contain user-defined parameters. Modules are indicated by individual cells. Cell body color indicates module input format, and cell outline color indicates module output format. Green = native file format; Blue = EEG in continuous array; Purple = EEG in segmented 3D array; Red = Output measure. For each module, sample parameters are outlined in black.

BEAPP is divided into four main steps, based on the format of input and output data (**Figure 1**). Step 1 involves converting native data into BEAPP format. Step 2 involves preprocessing of continuous data, for minimization of experimentally generated artifacts and standardization across acquisition setups. Step 3 involves dividing continuous data into segments for further analysis. Step 4 includes several options for analyses themselves, particularly those based on spectral decomposition. If users wish to input pre-segmented native data, they may skip steps 2 and 3 and proceed directly to analysis. Of note, while steps 1 and 2 are conceptually separate and thus described separately in the manuscript, within the GUI these steps are combined under the “Format and Preprocessing” option.

Within each step, the user can select any series of modules. Within modules, further customizable options and parameters are available. The user inputs offer a script-based or GUI-based menu to determine whether to include each given module, choose which options and parameters being used for that module, and decide whether to save the output of that module. Default parameters are offered to guide a beginning user, and as an example of parameter input format. Outputs include EEG data produced in that module, along with file-specific and group-level information intended to allow a user to easily keep track of how raw data have been altered, while avoiding unnecessary redundancy that may lead to excessively large file sizes.

The user guide contains detailed information about how to prepare EEG data to be run through BEAPP, including a “Start-Up Guide” for preparing an initial data run. This includes information on ensuring a user has the software necessary for running BEAPP, preparing the EEG files that will be analyzed (in a format recognizable by BEAPP), specifying any additional file-specific information not contained within the original EEG files themselves (e.g., line noise frequency), and setting the modules (and the options and parameters within these modules) to be run on the data. There is also a BEAPP “Module Creation Starter Guide” provided with the BEAPP software, to help users add new functionality.

### Step 1: Formatting Data for BEAPP

Prior to running any processing, segmenting, or analysis modules, BEAPP converts data from its native file format into BEAPP file format. BEAPP can handle several native file formats; details about how to prepare native files are included in the user guide. BEAPP format includes a cell array labeled *eeg*, which contains one matrix per recording period with the relevant EEG data. Each row of the matrix contains the amplitude of the EEG tracing for a particular channel, and each column contains the amplitude of the EEG tracing at a particular timepoint. For example, data obtained from an EEG with 129 channels over 60 s, sampled at 500 Hz, would be a 129 × 30,000 matrix. BEAPP format also includes a variable labeled *file_proc_info*, which contains key information specific to a particular EEG, including electrode layout, event timing, and sampling rate. As each file runs through the BEAPP platform, details of what processing steps the EEG has undergone are also included in *file_proc_info*. Of note, files in BEAPP format can be converted to and from EEGLAB file format as needed, using the batch_beapp2eeglab.m and batch_eeglab2beapp.m functions, respectively.

Native continuous (unsegmented) EEG data, with tags defining any events for later segmentation if necessary, provides the greatest flexibility in terms of which modules can be run in BEAPP. However, if users wish to provide preprocessed, pre-segmented data to BEAPP for analysis, this can be accommodated as well.

The formatting module requires users to provide BEAPP with the native EEG data and additional necessary information, including information about data type (baseline, event-tagged, or conditioned baseline). For our purposes, “baseline” refers to data that is continuously collected and not tied to any particular stimulus or time point. (If any event tags happen to exist in data marked as “baseline,” these tags will be ignored in the analysis and will not determine how the data are segmented). For many users, resting-state data may fit these criteria. “Event-tagged” refers to data that, while perhaps continuously collected, contains tags that specify a particular stimulus or time point around which segmentation should occur. This can be used for event-related potential (ERP) paradigms, for example. “Conditioned baseline” refers to a hybrid of baseline and event-tagged data, in which baseline data occurs between event tags; this includes alternating or recurring sections of baseline data. Resting data in which the eyes are intermittently opened and closed, sleep stages, or data containing intermittent epileptiform activity would often be of this data type. There is also an option to specify whether particular “recording periods” of data should be analyzed. In BEAPP, “recording periods” refer to sections of continuous data in a non-continuous data file. For example, if an EEG file contains one minute of continuous data, followed by a break during which no EEG was recorded, and then another minute of continuous data, then each of these one-minute runs of continuous data would be considered a recording period, and outputs would be reported separately for each recording period. Additionally, users provide information about electrode layout(s), line noise frequency, sampling rate(s), event tag name(s), and event tag offset(s), all of which may differ across EEGs within a single dataset.

### Step 2: Preprocessing of Continuous Data

In step 2 (preprocessing of continuous data), each module involves input of a continuous EEG signal, and output of another continuous EEG signal after modification by that module. The first module the user selects utilizes the user-provided EEG as its input, and each successive module that the user selects builds upon changes to the EEG made in the prior module. As above, the user can determine which modules to use, and which to turn off.

#### PREP Pipeline

In step 2, the first module offered is the PREP pipeline (Bigdely-Shamlo et al., 2015). The PREP pipeline is offered first because it was developed as a standardized early-stage preprocessing pipeline, including line noise removal using the *cleanline* method (Mullen, 2012), and robust average referencing with detection and interpolation of bad channels relative to this reference. Because line noise frequency varies by region (60 Hz in most of North America, 50 Hz in most of Europe, Africa, and Australia, and either 50 or 60 Hz in parts of South America and Asia), the BEAPP user is offered the opportunity to specify expected line noise frequency for their dataset in the formatting module, and this is altered accordingly in PREP. For users who may wish to evaluate data from multiple countries with potentially different line noise frequencies, users also have the option to define line noise frequency separately for each individual file. Since PREP is intended to be a standardized pipeline, optional input parameters for PREP through BEAPP are otherwise intentionally limited, although an advanced user could manually make changes directly within PREP as needed. Outputs include information about a variety of variables determined by the PREP pipeline, including whether any errors were encountered, and which channels PREP interpolated.

#### Filtering

Users may next use the filter module to apply their choice of high pass, low pass, and/or notch filtering steps, and can set the frequency parameters for each of these. BEAPP currently uses the EEGLAB *eegfiltnew* function for the high pass, low pass, and notch filters in this module, pulling in file-specific EEG data and sampling rate. *Cleanline* (Mullen, 2012) is also available for line noise removal.

Several checks are included in this module. First, since the user is offered the opportunity to later resample after filtering, BEAPP verifies that the minimum allowed sampling rate (after resampling) will not be less than twice the maximum frequency of the low pass filter, in order to avoid aliasing during resampling. (Of note, given imperfections in a typical filter, we would in fact recommend that the low pass filter be set below, rather than at, the Nyquist frequency of the resampling rate). Alternatively, if data in a particular file is sampled at a rate lower than the maximum good frequency in the low pass filter, filtering is skipped for that file. User notifications are generated in both of these cases, although the pipeline will continue running.

Typically a user will opt to remove line noise using either a notch filter or *cleanline*, but not both. Of note, *cleanline* is applied in both the PREP pipeline and the HAPPE pipeline (part of the ICA module, and described in companion paper; Gabard-Durnam et al., 2018); therefore, users applying PREP or HAPPE to their data will likely opt to turn both notch filtering and *cleanline* steps off in this module. By default, if the notch filter or *cleanline* are turned on, they will target frequency components in the range of the user-defined line noise.

#### Resampling

The next module offered is resampling. Users are currently offered the option to resample using interpolation via the MATLAB *interp1* function. While resampling can be used for any number of reasons, in BEAPP its primary utility is likely to standardize sampling rates across multiple acquisition setups (and hence potentially multiple acquisition sampling rates), by downsampling those EEGs collected at higher sampling rates to match the EEGS collected at lower sampling rates.

#### Independent Components Analysis (ICA)

BEAPP includes an ICA module, which provides three options for applying ICA to a dataset. One option is ICA alone, which decomposes the data from selected channels into a series of components maximizing temporal independence from one another. BEAPP employs the extended infomax ICA algorithm (with pre-whitening) to account for sources with subgaussian or supergaussian activity distributions (Lee et al., 1999). Relative to other ICA algorithms and decomposition methods, this ICA algorithm has been shown to be useful for decomposing electrophysiological signals like EEG (Delorme et al., 2007). If the user chooses this option, the EEG run through the remainder of BEAPP will include these components in place of channels, although the format (and variable names) will otherwise remain unchanged. Users may choose this option if they wish to analyze a particular component or series of components.

As a second option, if users wish to use ICA for artifact rejection, they may choose the option for ICA with a multiple artifact rejection algorithm (MARA). While details of MARA are described elsewhere (Winkler et al., 2011, 2014), it is worth noting that MARA has been shown to identify multiple types of artifact (including that from muscle and eye movements) for rejection in an automated manner, and can be applied to different electrode placements. While other options for automated artifact detection could be added in the future, we began with MARA because of its fully automated approach, its generalizability across participants and EEG acquisitions, and its ability to detect multiple classes of artifacts (rather than being restricted to a single artifact type). For users who wish to visualize the components that MARA selects for rejection, or manually select alternative components for rejection, a visualization option is provided; however, users should note that this addition of a manual step may decrease reproducibility of the otherwise automated EEG processing in BEAPP.

Of note, although there is still much empirical research to be done on processing steps and data parameters to optimize ICA performance (e.g., filtering settings, data quality, downsampling), ICA works best under several conditions. ICA performs optimally when data has been high pass filtered to remove non-stationary signal, and low-pass filtered to remove frequencies outside the range of biological sources (e.g., 250 Hz by some accounts) (Winkler et al., 2014). To avoid sensitivity to slow drift, high pass filtering at 0.5 Hz, or up to a maximum 2 Hz, is also recommended. While empirical testing is limited, it is also often recommended that for a given number of channels *c* and number of data samples *s*:

$$s\geq x*c^{2}$$

where *x* is a minimum of 20–30. This is to avoid overlearning and generate a robust, stable ICA signal decomposition (Särelä and Vigário, 2003). Users should take into account any down-sampling from the raw data when calculating their (effective) data samples. A large number of data samples and seconds of EEG data are necessary but not sufficient to guarantee robust ICA decomposition; therefore, these may serve as preliminary guidelines in combination with the abovementioned parameters.

MARA works best with epochs that are at least 15 s long, and suboptimal results may occur if data is high pass filtered at frequencies greater than 2 Hz or low pass filtered at frequencies less than 39 Hz. MARA calculates several metrics as part of its artifact detection algorithm that use spatial information from the 10–20 electrodes (or their equivalents), so users wishing to apply MARA should have channel locations with known 10–20 channel equivalents. Accordingly, within BEAPP, ICA/MARA currently runs on any channels in the acquisition layout whose placement corresponds to an electrode in the 10–20 system, and on any additional channels defined by the user to achieve the relationship between channels and samples as described above.

As a third option (and, if the user chooses, as an alternative to the other modules in step 2), the user also has the opportunity to run the HAPPE pipeline within BEAPP. HAPPE is described in a companion manuscript to this one (Gabard-Durnam et al., 2018), and is targeted toward artifact removal for EEG data collected from young children, those with neurodevelopmental disorders, or other EEG data with short recording lengths or high levels of artifact contamination. HAPPE includes highpass filtering at 1 Hz (bandpass filtering 1–249 Hz for EEGs sampled at 500 Hz or higher), *cleanline* for line noise removal, automated bad channel detection and removal, wavelet-enhanced ICA (W-ICA) and ICA with MARA component rejection for artifact removal, interpolation of bad channels, and referencing to average or single-channel or channel subset references. In the segmentation step of BEAPP, HAPPE also offers specific segmentation options, including automated segment rejection. HAPPE produces a single summary report across all EEGs of data quality metrics for each EEG to facilitate evaluation of files for inclusion in further analysis and to assess HAPPE performance on the data. For further details, see the companion paper on HAPPE (Gabard-Durnam et al., 2018).

#### Re-referencing

BEAPP then offers several options for re-referencing data. One referencing option is the infinity reference obtained by the reference electrode standardization technique (REST) (Yao, 2001, Yao et al., 2005; Dong et al., 2017), which is provided since multiple studies have recently found performance of REST to be superior to other referencing techniques (Chella et al., 2017; Huang et al., 2017; Lei and Liao, 2017; Liang et al., 2017).

If users prefer, data can be referenced to average at this stage. (Of note, average referencing does not need to be repeated at this stage if native data was average referenced, or if either PREP or HAPPE were already run on a dataset, because these pipelines output average referenced data). Data can also be referenced to a single channel or user-defined channel subset. Alternatively, data can be Laplacian referenced, using the CSD toolbox (Kayser and Tenke, 2006a). The Laplacian transform is well-regarded by many signal processors for its ability to help counteract the negative effects of volume conduction and recording reference (Kayser and Tenke, 2015) as well as muscle artifact (Fitzgibbon et al., 2013); additionally, Laplacian and average referencing can provide complementary information, as these techniques allow for targeted analysis of localized and widespread activity, respectively (Levin et al., 2017b).

There is a complex relationship between referencing and channel interpolation. A channel with poor signal quality (e.g., an electrode that was not appropriately attached to the head) can significantly alter the appearance not only of its own tracing, but also the tracing of any channels referenced to it. Therefore, such channels are often interpolated prior to re-referencing. However, interpolation itself depends on data in the surrounding channels; if more than one channel has poor signal quality, signal in an interpolated channel may have persistently poor quality. Additionally, determination of which channels have poor signal quality may differ depending upon the reference type being used.

To address this concern, BEAPP offers several options. As one option, users may opt to use the PREP pipeline (Bigdely-Shamlo et al., 2015), which includes a robust re-referencing procedure. This procedure involves iterative estimation of the average referenced signal, identification and interpolation of bad channels relative to this average referenced signal, and then re-estimation of the average referenced signal. This process is continued until iteratively alternating between these two processes no longer changes which channels are identified as requiring interpolation. Alternatively, users may opt to use ICA with MARA or HAPPE prior to re-referencing; in both of these options, for high-density EEG only a subset of the original channels are typically maintained (given limitations on the relationship between number of channels, which informs the number of ICA components, and number of data samples) (Makeig and Onton, 2011). Within this subset of channels, HAPPE identifies channels with poor signal quality and removes them before running ICA. ICA with MARA (either on its own or as part of HAPPE) then essentially acts upon remaining channels in the user-defined subset to minimize artifact while maintaining underlying signal. Any channels within the subset that had been removed earlier in processing are then interpolated. After PREP, ICA with MARA, or HAPPE, users can choose either to maintain any interpolated channels as they are, or remove any interpolated channels (substituting their data with NaN) so that they are not included in re-referencing and further steps.

#### Detrending

The final module currently offered in the preprocessing step of BEAPP is a detrending option. Currently users may choose mean, linear, or Kalman detrending. While mean or linear detrending will likely suffice for most users, Kalman detrending has been used for removal of artifact from transcranial magnetic stimulation (Morbidi et al., 2008) and ballistocardiogram (In et al., 2006), as well as epileptic spike detection (Tzallas et al., 2006). Kalman filtering works within a Bayesian framework, uses surrounding information to estimate the state of a process, and thus estimate the noise to be removed from the signal. (Kalman, 1960). Notably, “detrending” in this module refers to processing of the continuous EEG. A separate option for detrending within individual segments is also available in step 3.

### Step 3: Division of Data Into Segments

In step 3 (division of data into segments), the input is a continuous EEG signal. This is typically the last EEG signal output from step 2 after preprocessing is complete, although users who have preprocessed their data outside of BEAPP may choose to proceed directly to step 3 after formatting. The output is a series of EEG segments, which are ready for the analyses offered in step 4.

During the segmentation step, users may specify whether to treat data as baseline data, event-related data, or conditioned baseline data and whether any additional processing (e.g., within-segment detrending) takes place. Additionally, this step allows the user to provide rejection criteria for each segment.

#### Event-Related Data

For event-related EEG, the data segments created are time-locked to a stimulus or other marked event (hereafter “stimulus” will refer to all cases of an event). The user can define the event code for this stimulus of interest, and specify segment start and end times in relation to the stimulus. Start and end times can be negative or positive, for situations in which the user would like segments to include data before or after stimulus offset, respectively. If the timing of the event code is offset from the true stimulus delivery time (e.g., due to transmission delays in the stimulus presentation setup), the user can define this offset in the formatting module. If offsets are not uniform across the dataset, the user can provide a table that defines the offset for each individual file. Once segments are created, the user can choose whether to run a within-segment linear detrend. “Bad” segments can be rejected if data in any channel crosses an amplitude threshold set by the user, or using rejection criteria defined in HAPPE, which includes both amplitude thresholding and assessment of segment likelihood (where artifact-contaminated segments should be less likely than good segments) using joint probability calculations (both across segments for a single channel and across channels for a single segment).

Of note, this initial creation of segments is used primarily for within-segment preprocessing, and for determining which segments to reject. The user is not obligated to analyze all of the data within a segment once a segment has been created, however. In the analysis step (described below), the user can choose a sub-segment upon which to focus their analyses. This may be particularly useful for users who wish to baseline correct event-related data to a pre-stimulus baseline, or users who wish to run separate analyses on a series of sub-segments (e.g., early and late responses to a stimulus).

#### Baseline Data

**FIGURE 2:**
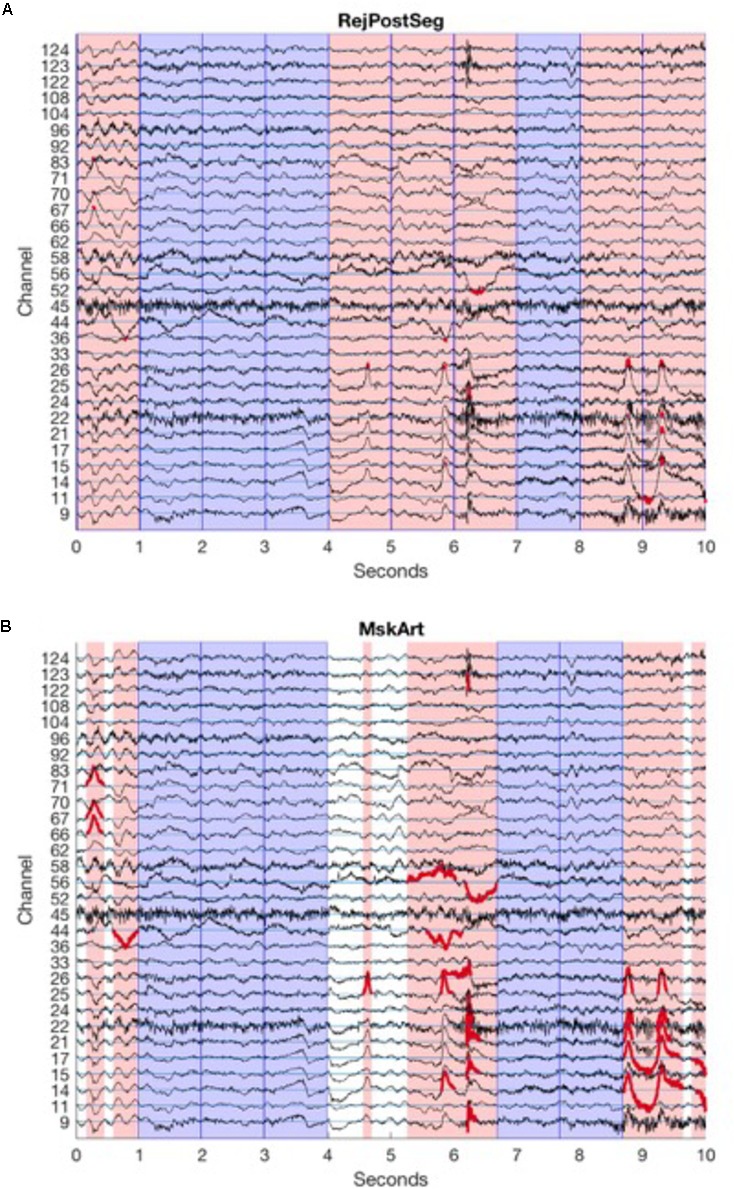
Segmentation and amplitude thresholding. Channels are arranged vertically, with horizontal channel reference lines in cyan. For clarity, only those channels that are part of the 10–20 system, and those channels that cross the amplitude threshold in this portion of the EEG, are shown here. EEG tracings for each channel are in black. Reference lines are spaced by 100 μV, equal to amplitude threshold, so that above-amplitude portions of tracing cross another channel’s reference line. Dark blue vertical lines indicate segment boundaries; in this example, 1 s segments are created from continuous data. Sample data here are from baselineEEG08.mat, samples 13,250:15,750, after PREP, filtering (1 Hz high pass, 100 Hz low pass), and mean detrending. **(A)** Amplitude thresholding after segmentation. Pink patches demonstrate segments rejected because at least one channel crosses the artifact threshold. Portions of the EEG tracing that are above the amplitude threshold (and thus lead a segment to be rejected) are in red. Blue patches demonstrate good segments to be analyzed. **(B)** Amplitude thresholding before segmentation. Pink patches demonstrate segments rejected because at least one channel crosses the artifact threshold; length of segment spans from zero-crossing before the segment crosses the artifact threshold to zero-crossing after the segment crosses the artifact threshold. Portions of the EEG tracing that are above the amplitude threshold, extending out to the zero-crossing on either side (thus determining the boundaries for rejected segments) are in red. Blue patches demonstrate good segments to be analyzed. White patches demonstrate remaining segments of good data that are too short for analysis.

For baseline data, the user defines the length of segments to be created, and BEAPP creates a series of non-overlapping segments from the available data. Amplitude thresholding or HAPPE-based thresholding for segment rejection can occur after BEAPP divides the full EEG into segments, in a manner identical to event-related segment rejection (**Figure 2A**). Alternatively, BEAPP offers an option to use amplitude thresholding to first identify segments of unusable data within the continuous EEG, and then create segments from the remaining data. For this purpose, BEAPP’s definition of unusable data is intended to closely match common hand-editing practices, which aim to identify high-amplitude data and its surrounding rise and fall. Upon identifying any data point that is above threshold, BEAPP determines the nearest zero-crossing before and after that data point. Above-threshold segments are then defined as beginning and ending at the nearest zero-crossings, rather than only including the narrower windows of time where data is suprathreshold. BEAPP creates a mask marking segments of data in which any channel is above threshold (as defined by the zero-crossing start and end points), and then creates segments from the remaining data (**Figure 2B**).

Regardless of whether segment rejection occurs before or after segmentation, non-continuous data are not concatenated in any way, given concerns about data integrity with concatenation. Data is divided into the maximum possible number of segments of user-specified length, but any remaining data at the end of each useable segment, if it is too short to create its own segment, is excluded from analysis.

#### Conditioned Baseline Data

As described above, conditioned baseline data is essentially a hybrid of baseline and event-tagged data, in which baseline data occurs between event tags. The user specifies the event tags that signify onset and offset of conditioned baseline periods. Then, as for baseline data, the user defines the length of segments to be created, and BEAPP segments the data between event tags accordingly.

#### Further Processing of Segments

Once segments have been created, BEAPP also offers an option for within-segment detrending. All of the analyses described below can then be run on a segment in its entirety, or on a sub-segment. For event-related data, users have the option to baseline correct one portion of the segment (typically a post-stimulus portion) to another portion of the segment (typically a pre-stimulus portion) prior to running analyses on a sub-segment.

### Step 4: Analyses

In step 4 (analyses), the inputs are the segmented EEG. Outputs are the results of analyses themselves. For each file, detailed outputs are reported in MATLAB, for users who wish to run further analyses on their own. Additionally, several summary outputs are output into a .csv file, for users who do not desire any further post-processing. Several basic analyses are currently offered in BEAPP. Users may also use the preprocessed data output from step 2 or segmented data output from step 3 as a basis for performing other analyses outside of BEAPP, if they prefer.

#### Power Spectrum

Spectral power is initially calculated on each segment or sub-segment created as above. Several options are offered for a how a power spectrum is calculated.

If the user opts to use a rectangular window or Hanning window, power is calculated by Fast Fourier Transform, using the *fft* function (Frigo and Johnson, 1998) in MATLAB. Each segment is zero-padded with trailing zeros to the nearest power of 2, as recommended by MATLAB to increase the performance of *fft* when the number of samples in a given segment is not a power of 2. This creates a double-sided power spectrum, with complex fft values. For a single side of this spectrum *eeg_wf*, absolute power *eeg_wfp* is then calculated for each segment, for given sampling rate *sr* and segment length (in samples) *l*, as follows:

$$eeg\_wfp=\frac{2*|eeg\_wf{|}^{2}}{l*sr}$$

If the user opts to use a multitaper method (Thomson, 1982) to calculate the power spectrum, they can choose the number of tapers to apply. The power spectrum is then calculated using the MATLAB *pmtm* function.

#### Inter-Trial Phase Coherence

Inter-trial phase coherence (ITPC), a measure of the extent to which an EEG signal is phase-locked to repeated time-locked events (Makeig et al., 2004), can be calculated across segments as well. This calculation uses the EEGLAB *newtimef.m* function. For each event condition and each channel, phase coherence is calculated across all segments, across relevant spectral time windows (determined by user-defined subwindow length) and frequency bins. Outputs of this module are complex numbers, which include information about ITPC magnitude (absolute value of the complex number) and phase (angle of the complex number).

### Summary Outputs

After each run of one or more files across one or more modules, BEAPP creates a folder called “out,” which contains detailed information summarizing the run. This includes a MATLAB structure specifying the user inputs and run settings that were common to all files included in a given run; of note, maintaining this information in its own file, rather than repeated within each individual EEG file, helps with management of data size (see below). There is also a file detailing the text output from the command window. Summary information is included in this folder for certain modules as well. For example, a .csv file contains summary information about files run through PREP, including whether any errors occurred and how many bad channels PREP detected. A separate .csv file contains summary information generated through HAPPE.

The output folder also includes summary information about analyses conducted. For analyses in the frequency domain, data can be binned into any number of frequency bands defined by the user. For power analyses, optional outputs in .csv format include mean power across all segments in each channel at each frequency band, as well as absolute power, normalized power, natural log of power, and log10 of power. For ITPC analyses, .csv outputs can include the maximum and/or mean ITPC magnitude for each channel. Analysis-generated .csv outputs also include general information relevant to interpretation of these outputs, such as the original EEG’s net type (EEG electrode layout), original sampling rate, current sampling rate (for resampled data), information about recording periods run (for original EEGs that included multiple recording periods), information about channels marked bad, and number of data segments analyzed.

### Management of Data Size

When running large EEG datasets and saving outputs at multiple processing modules, the amount of data being generated may rapidly become a burden. BEAPP addresses this concern in several ways. First, users are given the option of whether to save output of each module. If a user does not intend to review outputs of a specific module, or if outputs of a given module can be rapidly reproduced if needed in the future, users may choose to have BEAPP automatically delete these outputs. Outputs aim to strike a balance between maintaining adequate information to allow a user to easily keep track of how raw data have been altered and avoid unnecessary redundancy that may lead to excessively large file sizes.

### Sample Data Files

The specific examples in this manuscript come from application of BEAPP to EEG data collected through the Infant Sibling Project (ISP), a prospective investigation examining infants at high versus low familial risk for autism spectrum disorder over the first 3 years of life. This dataset was chosen because the longitudinal nature of the study led to data collection with different sampling rates (250 and 500 Hz) and acquisition setups (64-channel Geodesic Sensor Net v2.0, and 128-channel HydroCel Geodesic Sensor Net, both from Electrical Geodesics, Inc., Eugene, OR, United States). Additionally, because young children cannot follow instructions to “rest” or remain still, EEG in these children typically contains greater amounts of artifact than EEG in typical adults. Baseline EEG data was collected while a young child sat in a parent’s lap watching a research assistant blow bubbles or show toys (Levin et al., 2017b). Event-related EEG data was collected using an auditory double oddball paradigm, in which a stream of consonant-vowel stimuli was presented. Stimuli included a “Standard” /aaada/ sound 80% of the time, “Native” /ta/ sound 10% of the time, and “Non-Native” /da/ sound 10% of the time (Seery et al., 2014). To demonstrate applications of BEAPP to analysis of group data, a subset of the full ISP dataset, containing 10 baseline EEGs and 10 event-related EEGs, is available at zenodo.org (Levin et al., 2017a) Details regarding the baseline EEGs are provided in **Table 1**, and details regarding the event-related EEGs are provided in **Table 2**. Data were collected in the United States, with 60 Hz line noise. This study was carried out in accordance with the recommendations of the Institutional Review Board at Boston University and Boston Children’s Hospital, with written informed consent from all caregivers prior to their child’s participation in the study.

**Table 1:** Baseline EEG information.

File name	Sampling	Net type
	rate	
“baselineEEG01.mat”	250	“HydroCel GSN 128 1.0”
“baselineEEG02.mat”	250	“Geodesic Sensor Net 64 2.0”
“baselineEEG03.mat”	250	“Geodesic Sensor Net 64 2.0”
“baselineEEG04.mat”	250	“HydroCel GSN 128 1.0”
“baselineEEG05.mat”	250	“HydroCel GSN 128 1.0”
“baselineEEG06.mat”	250	“HydroCel GSN 128 1.0”
“baselineEEG07.mat”	250	“HydroCel GSN 128 1.0”
“baselineEEG08.mat”	500	“HydroCel GSN 128 1.0”
“baselineEEG09.mat”	500	“HydroCel GSN 128 1.0”
“baselineEEG10.mat”	500	“HydroCel GSN 128 1.0”


**Table 2:** Event-tagged EEG information.

File name	Sampling	Net type	Offset
	rate		
“auditoryEEG01.mff”	250	“HydroCel GSN 128 1.0”	0
“auditoryEEG02.mff”	250	“Geodesic Sensor Net 64 2.0”	0
“auditoryEEG03.mff”	250	“Geodesic Sensor Net 64 2.0”	0
“auditoryEEG04.mff”	250	“HydroCel GSN 128 1.0”	0
“auditoryEEG05.mff”	250	“HydroCel GSN 128 1.0”	0
“auditoryEEG06.mff”	250	“HydroCel GSN 128 1.0”	0
“auditoryEEG07.mff”	250	“HydroCel GSN 128 1.0”	8
“auditoryEEG08.mff”	500	“HydroCel GSN 128 1.0”	18
“auditoryEEG09.mff”	500	“HydroCel GSN 128 1.0”	18
“auditoryEEG10.mff”	500	“HydroCel GSN 128 1.0”	18


## Results

Data were run on a standard iMac (late 2015, 4 GHz Intel Core i7 processor, macOS X El Capitan version 10.11.6). BEAPP was initially created and tested in MATLAB 2016a, although data here were run using MATLAB 2017b.

### Testing BEAPP on a Sample Dataset: Baseline DATA, Power Spectrum

We ran BEAPP on a sample dataset of 10 baseline EEGs, as described above. Data ran through BEAPP for a variety of module combinations. A **Supplementary File 1** describing the exact settings for each of these analyses is included with this manuscript. Of note, the formatting and PREP modules were only run once each; the order of runs described above and in the table allowed subsequent runs to take advantage of these modules from prior runs.

**FIGURE 3:**
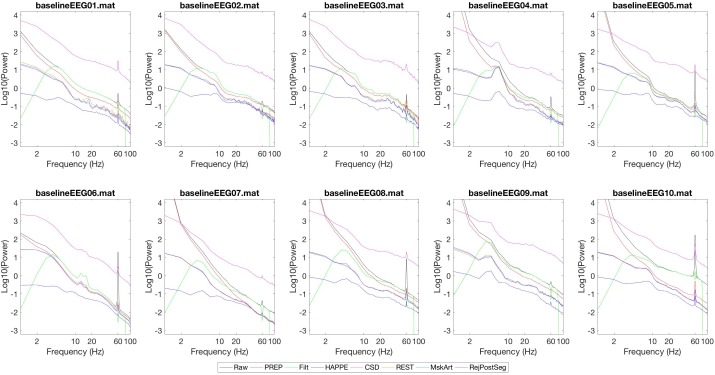
Power spectra generated by various preprocessing pipelines. Power spectra for each continuous EEG are shown on a log10-log10 scale. Power units are in μV^2^, except CSD (pink) which is in μV^2^/mm^2^. Each line shows the mean power spectrum generated across all channels. After specified processing, data were segmented into 1 s windows. No additional artifact rejection occurred prior to power analysis, unless otherwise specified. Black: Raw data. Red: PREP pipeline. Green: “Filt.” (Filtering 4 Hz high pass, 80 Hz low pass, 60 Hz notch). Dark blue: HAPPE pipeline (Filtering 1–100 Hz, resampling to 250 Hz, HAPPE pipeline with associated segment rejection). Pink: CSD (PREP pipeline, filtering 1–100 Hz, CSD rereferencing, mean detrending, artifact thresholding at 3,000 μV^2^/mm^2^ prior to segmentation). Orange: REST (PREP pipeline, filtering 1–100 Hz, REST rereferencing, mean detrending, artifact thresholding at 100 μV^2^ prior to segmentation). Light blue: MskArt (PREP pipeline, filtering 1–100 Hz, resampling to 250 Hz, mean detrending, artifact thresholding at 100 μV^2^ prior to segmentation). Purple: Same settings as MskArt, but with artifact thresholding after segmentation.

Power spectra generated with each of these configurations are shown in **Figure 3**. Power from the “Raw” data (after segmentation into 1 s segments without artifact rejection) is shown in black. After the PREP pipeline, which involves *cleanline* and robust average referencing, 60 Hz line noise is reduced (though not fully eliminated), and power is reduced across frequencies (red). After the “Filt” run (green), there is a reduction in power below 4 Hz with some extension into higher frequencies, due to the 4 Hz high pass filter. There is a reduction in power above 80 Hz, due to the 80 Hz low pass filter. There is a reduction in power at and near 60 Hz, due to the 60 Hz notch filter. After the HAPPE run (dark blue), there is a significant reduction in power across all frequencies as is expected with HAPPE preprocessing (Gabard-Durnam et al., 2018), reduction in 60 Hz line noise power (since HAPPE includes *cleanline*), and some reduction in power just above 1 Hz and just below 100 Hz, due to high and low pass filters respectively occurring at these frequencies. With CSD rereferencing (pink) after the PREP pipeline and 1–100 Hz filtering, the power spectrum takes on the shape expected from these preprocessing steps, but with increased power across frequencies overall. (Of note, power units for CSD are μV^2^/mm^2^, whereas power units are otherwise in μV^2^). MskArt (light blue) and RejPostSeg (purple), with artifact thresholding taking place before and after segmentation respectively, give relatively similar results to one another; REST (orange) also gives similar results.

While the exact amount of time each step requires will depend on multiple factors (e.g., file size, computer specifications, etc.), approximate benchmarks for the computer described above are provided here for transparency. On average for each file, modules took the following amounts of time: Format <10 s, PREP 1.5 min, filter <10 s, resample <10 s, HAPPE 3.5 min, CSD 30 s, REST <10 s, detrend <10 s, segment <10 s, PSD <10 s.

### Testing BEAPP on a Sample Dataset: Event-Tagged Data, ITPC

**FIGURE 4:**
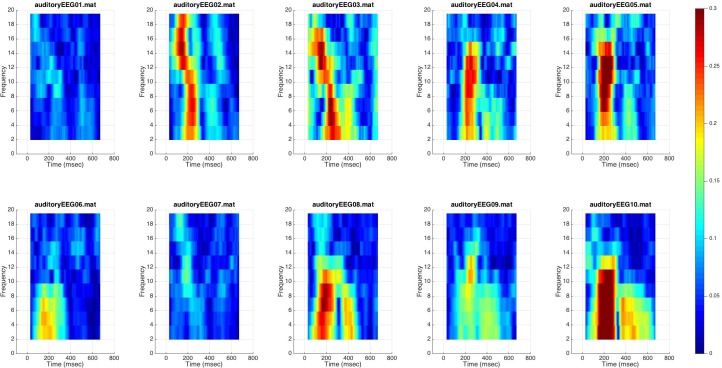
Inter-trial phase coherence for event-tagged data. ITPC for each event-tagged EEG is shown on a surface plot. Color demonstrates magnitude of ITPC at each time window, for each frequency band. ITPC for the frontocentral channel demonstrating maximum ITPC (across all time windows centered 100–300 ms after stimulus onset, and across all frequency bands) is plotted for each EEG; thus each plot contains data from only one channel.

We next ran BEAPP on the sample dataset of 10 event-tagged EEGs. Processing steps after formatting included the PREP pipeline, filtering 1–100 Hz, resampling to 250 Hz, CSD rereferencing, mean detrending, segmenting from -100 to 800 ms in relationship to the event tag for each “Standard” stimulus (taking into account system offsets, which had not been accounted for in the original event tags), and evaluation ITPC across multiple overlapping windows of 256 ms each, and across multiple frequency bands. On average, the ITPC module took 30 s per file. **Figure 4** plots the outcome of this analysis for the frontocentral channel demonstrating maximum ITPC in each EEG. There is a peak in ITPC in the 150–300 ms time windows, across multiple frequencies, for most of the sample EEGs. Of note, this finding overlaps exactly with the time windows in which one expects a positive-going P150 component in event-related potential (ERP) analyses of young children (Seery et al., 2014).

## Discussion

BEAPP automates batch EEG processing and analyses across multiple EEGs (including those collected across multiple acquisition setups) and multiple processing steps. Rather than prescribing a specified set of processing steps, BEAPP allows users to choose from a menu of options that meet their needs.

BEAPP has two primary goals. The first is accessibility. BEAPP aims to provide a bridge, allowing researchers studying the brain to more easily access some of the most useful tools that experienced signal processors have created, by replicating others’ analysis pipelines or creating their own. To this end, BEAPP aims to strike a balance between assuming only a basic level of MATLAB and EEG signal processing experience, while also offering a flexible menu of opportunities for more advanced users.

The second primary goal is reproducibility. By allowing improved accessibility of methods and workflows across execution environments, BEAPP aims to improve reproducibility within experiments, replicability across experiments, and collaboration across laboratories.

Several limitations of BEAPP merit discussion. First, while BEAPP provides flexibility for running a variety of modules with a variety of user-specified parameters, BEAPP does not prevent an inexperienced user from running incorrect sequences of analyses, or setting meaningless parameters. For example, a user running the PREP or HAPPE pipelines within BEAPP will need to have adequate knowledge of these pipelines (either through reading their companion manuscripts, or through the BEAPP user guide) to know that these pipelines include *cleanline* and average referencing; therefore, such steps do not need to be run separately in other modules. As another example, running ITPC on baseline data would produce outputs, but the meaning of such results (since data segments would not be tied to any particular stimulus) would be questionable. In many cases, tips to avoid such errors are included in the user guide, or within the user inputs themselves. BEAPP also contains flags to help avoid certain errors (such as unintentionally overwriting previously generated data when running modules with a new set of parameters). However, as is often the case with customizable software, such errors are not fully avoidable.

BEAPP is currently also limited in the scope of processing techniques that it offers. The processing algorithms contained in other software packages are numerous and tremendously useful, and BEAPP incorporates only a small subset of the tools available. In its current form, BEAPP therefore acts predominantly as a basic structure for EEG data analysis and tracking, upon which other parameters, modules, and options can be added in the future. Future additions to BEAPP would likely include additional options for managing a wider variety of native file types, compatibility with the Brain Imaging Data Structure (BIDS) (Gorgolewski et al., 2016), improved efficiency through parallel processing, additional analysis options (e.g., coherence, phase lag index, and phase amplitude coupling), and display options (e.g., topoplots). Rather than a fixed pipeline, BEAPP is intended to offer a framework that a beginning user can use for batch EEG data processing, and upon which a more advanced user can build in additional options for preprocessing and analysis.

Overall, BEAPP aims to provide a structure to streamline batch processing of EEG across multiple preprocessing and analysis steps, and across multiple EEGs in a dataset (including EEGs with differing acquisition setups). Long term goals of this structure include improved accessibility to EEG analysis across fields, and improved reproducibility thereof.

## Author’s Note

BEAPP is copyright 2015, 2016, 2017, 2018. This program is free software: you can redistribute it and/or modify it under the terms of the GNU General Public License (version 3) as published by the Free Software Foundation.

## Author Contributions

AL conceived of BEAPP and drafted the manuscript. AL, AML, LG-D, and HO’L contributed and tested the code. HO’L focused predominantly on signal processing aspects of code, AML focused on code development, and LG-D focused on integration of the HAPPE pipeline into BEAPP (see associated manuscript). AL, AML, LG-D, and HO’L contributed to design of BEAPP, analysis and interpretation of data for BEAPP, critically revising the manuscript for important intellectual content, and approving the final version for submission.

## Conflict of Interest Statement

The authors declare that the research was conducted in the absence of any commercial or financial relationships that could be construed as a potential conflict of interest.

## References

[B1] Bigdely-Shamlo N. Mullen T. Kothe C. Su K.-M. Robbins K. A. (2015). The PREP pipeline: standardized preprocessing for large-scale EEG analysis. *Front. Neuroinform.* 9:16. 10.3389/fninf.2015.00016 26150785 PMC4471356

[B2] Chella F. D’Andrea A. Basti A. Pizzella V. Marzetti L. (2017). Non-linear analysis of scalp EEG by using bispectra: the effect of the reference choice. *Front. Neurosci.* 11:262. 10.3389/fnins.2017.00262 28559790 PMC5432555

[B3] Delorme A. Makeig S. (2004). EEGLAB: an open source toolbox for analysis of single-trial EEG dynamics including independent component analysis. *J. Neurosci. Methods* 134 9–21. 10.1016/j.jneumeth.2003.10.009 15102499

[B4] Delorme A. Palmer J. Oostenveld R. Onton J. Makeig S. (2007). *Comparing* *Results of Algorithms Implementing Blind Source Separation of EEG Data* Available at: https://sccn.ucsd.edu/tildearno/mypapers/delorme_unpub.pdf [Accessed September 24 2017].

[B5] Dong L. Li F. Liu Q. Wen X. Lai Y. Xu P. (2017). MATLAB toolboxes for reference electrode standardization technique (REST) of scalp EEG. *Front. Neurosci.* 11:601. 10.3389/fnins.2017.00601 29163006 PMC5670162

[B6] Fitzgibbon S. P. Lewis T. W. Powers D. M. W. Whitham E. W. Willoughby J. O. Pope K. J. (2013). Surface laplacian of central scalp electrical signals is insensitive to muscle contamination. *IEEE Trans. Biomed. Eng.* 60 4–9. 10.1109/TBME.2012.2195662 22542648

[B7] Free Software Foundation (2007). *GNU General Public License.* Available at: http://www.gnu.org/licenses/gpl.html

[B8] Frigo M. Johnson S. G. (1998). “FFTW: an adaptive software architecture for the FFT,” in Proceedings of the 1998 IEEE International Conference on Acoustics, Speech and Signal Processing, ICASSP ’98, (Seattle, WA: IEEE), 1381–1384. 10.1109/ICASSP.1998.681704

[B9] Gabard-Durnam L. J. Mendez Leal A. S. Wilkinson C. L. Levin A. R. (2018). The Harvard Automated Processing Pipeline for Electroencephalography (HAPPE): standardized processing software for developmental and high-artifact data. *Front. Neurosci.* 12:97. 10.3389/fnins.2018.00097 29535597 PMC5835235

[B10] Gorgolewski K. J. Auer T. Calhoun V. D. Craddock R. C. Das S. Duff E. P. (2016). The brain imaging data structure, a format for organizing and describing outputs of neuroimaging experiments. *Sci. Data* 3:160044. 10.1038/sdata.2016.44 27326542 PMC4978148

[B11] Gramfort A. (2013). MEG and EEG data analysis with MNE-Python. *Front. Neurosci.* 7:267. 10.3389/fnins.2013.00267 24431986 PMC3872725

[B12] Gramfort A. Luessi M. Larson E. Engemann D. A. Strohmeier D. Brodbeck C. (2014). MNE software for processing MEG and EEG data. *Neuroimage* 86 446–460. 10.1016/j.neuroimage.2013.10.027 24161808 PMC3930851

[B13] Huang Y. Zhang J. Cui Y. Yang G. Liu Q. He L. (2017). How different EEG references influence sensor level functional connectivity graphs. *Front. Neurosci.* 11:368. 10.3389/fnins.2017.00368 28725175 PMC5496954

[B14] In M. H. Lee S. Y. Park T. S. Kim T. S. Cho M. H. Ahn Y. B. (2006). Ballistocardiogram artifact removal from EEG signals using adaptive filtering of EOG signals. *Physiol. Meas.* 27 1227–1240. 10.1088/0967-3334/27/11/014 17028414

[B15] Kalman R. E. (1960). A new approach to linear filtering and prediction problems. *Trans. ASME J. Basic Eng.* 82 35–45. 10.1115/1.3662552

[B16] Kayser J. Tenke C. E. (2006a). Principal components analysis of laplacian waveforms as a generic method for identifying ERP generator patterns: I. Evaluation with auditory oddball tasks. *Clin. Neurophysiol.* 117 348–368. 10.1016/j.clinph.2005.08.034 16356767

[B17] Kayser J. Tenke C. E. (2006b). Principal components analysis of laplacian waveforms as a generic method for identifying ERP generator patterns: II. Adequacy of low-density estimates. *Clin. Neurophysiol.* 117 369–380. 10.1016/j.clinph.2005.08.033 16356768

[B18] Kayser J. Tenke C. E. (2015). Issues and considerations for using the scalp surface laplacian in EEG/ERP research: a tutorial review. *Int. J. Psychophysiol.* 97 189–209. 10.1016/j.ijpsycho.2015.04.012 25920962 PMC4537804

[B19] Lee T. W. Girolami M. Sejnowski T. J. (1999). Independent component analysis using an extended infomax algorithm for mixed subgaussian and supergaussian sources. *Neural Comput.* 11 417–441. 10.1162/089976699300016719 9950738

[B20] Lei X. Liao K. (2017). Understanding the influences of EEG reference: a large-scale brain network perspective. *Front. Neurosci.* 11:205. 10.3389/fnins.2017.00205 28450827 PMC5390022

[B21] Levin A. R. Gabard-Durnam L. J. Mendez Leal A. S. O’Leary H. M. Wilkinson C. L. Tager-Flusberg H. (2017a). *Infant Sibling Project: Sample Files.* 10.5281/zenodo.998964 Available at: https://zenodo.org/record/998965#.WdBg2BNSxBw

[B22] Levin A. R. Varcin K. J. O’Leary H. M. Tager-Flusberg H. Nelson C. A. (2017b). EEG power at 3 months in infants at high familial risk for autism. *J. Neurodev. Disord.* 9:34. 10.1186/s11689-017-9214-9 28903722 PMC5598007

[B23] Liang T. Hu Z. Li Y. Ye C. Liu Q. (2017). Electrophysiological correlates of change detection during delayed matching task: a comparison of different references. *Front. Neurosci.* 11:527. 10.3389/fnins.2017.00527 29018318 PMC5623019

[B24] Litvak V. Mattout J. Kiebel S. Phillips C. Henson R. Kilner J. (2011). EEG and MEG Data Analysis in SPM8. *Comput. Intell. Neurosci.* 2011:852961. 10.1155/2011/852961 21437221 PMC3061292

[B25] Makeig S. Debener S. Onton J. Delorme A. (2004). Mining event-related brain dynamics. *Trends Cogn. Sci.* 8 204–210. 10.1016/j.tics.2004.03.008 15120678

[B26] Makeig S. Onton J. (2011). “ERP features and EEG dynamics: an ICA perspective,” in *Oxfort Handbook of Event-Related Potential Components*. New York, NY: Oxford University Press 10.1093/oxfordhb/9780195374148.013.0035

[B27] Morbidi F. Garulli A. Prattichizzo D. Rizzo C. Rossi S. (2008). Application of kalman filter to remove TMS-induced artifacts from EEG recordings. *IEEE Trans. Control Syst. Technol.* 16 1360–1366. 10.1109/TCST.2008.921814

[B28] Mullen T. (2012). *NITRC: Cleanline.* San Diego, CA: Tool/Resource Info.

[B29] Oostenveld R. Fries P. Maris E. Schoffelen J.-M. (2011). FieldTrip: open source software for advanced analysis of MEG, EEG, and invasive electrophysiological data. *Comput. Intell. Neurosci.* 2011:156869. 10.1155/2011/156869 21253357 PMC3021840

[B30] Peng R. D. (2011). Reproducible research in computational science. *Science* 334 1226–1227. 10.1126/science.1213847 22144613 PMC3383002

[B31] Särelä J. Vigário R. (2003). Overlearning in marginal distribution-based ICA: analysis and solutions. *J. Mach. Learn. Res.* 4 1447–1469.

[B32] Seery A. Tager-Flusberg H. Nelson C. A. (2014). Event-related potentials to repeated speech in 9-month-old infants at risk for autism spectrum disorder. *J. Neurodev. Disord.* 6:43. 10.1186/1866-1955-6-43 25937843 PMC4416338

[B33] Tadel F. Baillet S. Mosher J. C. Pantazis D. Leahy R. M. (2011). Brainstorm: a user-friendly application for MEG/EEG analysis. *Comput. Intell. Neurosci.* 2011:879716. 10.1155/2011/879716 21584256 PMC3090754

[B34] Tannous B. A. Teng J. (2011). Secreted blood reporters: insights and applications. *Biotechnol. Adv.* 29 997–1003. 10.1016/j.biotechadv.2011.08.021 21920429 PMC3189544

[B35] Thomson D. J. (1982). Spectrum estimation and harmonic analysis. *Proc. IEEE* 70 1055–1096. 10.1109/PROC.1982.12433

[B36] Tzallas A. T. Oikonomou V. P. Fotiadis D. I. (2006). “Epileptic spike detection using a kalman filter based approach,” in Proceedings of the 28th IEEE EMBS Annual International Conference, (New York, NY), 501–504. 10.1109/IEMBS.2006.260780 17945981

[B37] Winkler I. Brandl S. Horn F. Waldburger E. Allefeld C. Tangermann M. (2014). Robust artifactual independent component classification for BCI practitioners. *J. Neural Eng.* 11:035013. 10.1088/1741-2560/11/3/035013 24836294

[B38] Winkler I. Haufe S. Tangermann M. (2011). Automatic classification of artifactual ICA-components for artifact removal in EEG signals. *Behav. Brain Funct.* 7:30. 10.1186/1744-9081-7-30 21810266 PMC3175453

[B39] Yao D. (2001). A method to standardize a reference of scalp EEG recordings to a point at infinity. *Physiol. Meas.* 22 693–711. 10.1088/0967-3334/22/4/305 11761077

[B40] Yao D. Wang L. Oostenveld R. Nielsen K. D. Arendt-Nielsen L. Chen A. C. N. (2005). A comparative study of different references for EEG spectral mapping: the issue of the neutral reference and the use of the infinity reference. *Physiol. Meas.* 26 173–184. 10.1088/0967-3334/26/3/003 15798293

