# Supplementary material for: BEAPP: The Batch Electroencephalography Automated Processing Platform
Source: Front Neurosci. 2018 Aug 7;12:513. doi: 10.3389/fnins.2018.00513 (PMC6090769; doi:10.3389/fnins.2018.00513)
Supplement: Supplementary file 1 [file Data_Sheet_1.PDF]

Supplemental File 1. BEAPP Inputs for figures 2, 3, and 4

	Resting								Auditory
	Raw	PREP	Filter	HAPPE	CSD	REST	MskArt	RejPostSeg	ITPC
grp_proc_info.src_dir	{'/Volumes/ISP/Resting'};[1]								{'/Volumes/ISP/Auditory'};[1]
grp_proc_info.beapp_curr_run_tag	'Raw';	'PREP';	'Filt';	'HAPPE';	'CSD';	'REST';	'MskArt';	'RejPostSeg';	'ITPC';
grp_proc_info.beapp_prev_run_tag[2]	'';	Raw';	Raw';	Raw';	PREP';	'PREP';	PREP';	PREP';	'';
grp_proc_info.beapp_advinputs_on	0;								0;
grp_proc_info.beapp_toggle_mods{'format',{'Module_On','Module_Export_On'}}	[1,1];	[0,0];	[0,0];	[0,0];	[0,0];	[0,0];	[0,0];	[0,0];	[1,1];
grp_proc_info.beapp_toggle_mods{'prepp',{'Module_On','Module_Export_On'}}	[0,0];	[1,1];	[0,0];	[0,0];	[0,0];	[0,0];	[0,0];	[0,0];	[1,1];
grp_proc_info.beapp_toggle_mods{'filt',{'Module_On','Module_Export_On'}}	[0,0];	[0,0];	[1,1];	[1,1];	[1,1];	[1,1];	[1,1];	[1,1];	[1,1];
grp_proc_info.beapp_toggle_mods{'rsamp',{'Module_On','Module_Export_On'}}	[0,0];	[0,0];	[0,0];	[1,1];	[0,0];	[0,0];	[1,1];	[1,1];	[1,1];
grp_proc_info.beapp_toggle_mods{'ica',{'Module_On','Module_Export_On'}}	[0,0];	[0,0];	[0,0];	[1,1];	[0,0];	[0,0];	[0,0];	[0,0];	[0,0];
grp_proc_info.beapp_toggle_mods{'rereference',{'Module_On','Module_Export_On'}}	[0,0];	[0,0];	[0,0];	[0,0];	[1,1];	[1,1];	[0,0];	[0,0];	[1,1];
grp_proc_info.beapp_toggle_mods{'detrend',{'Module_On','Module_Export_On'}}	[0,0];	[0,0];	[0,0];	[0,0];	[1,1];	[1,1];	[1,1];	[1,1];	[1,1];
grp_proc_info.beapp_toggle_mods{'segment',{'Module_On','Module_Export_On'}}	[1,1];	[1,1];	[1,1];	[1,1];	[1,1];	[1,1];	[1,1];	[1,1];	[1,1];
grp_proc_info.beapp_toggle_mods{'psd',{'Module_On','Module_Export_On'}}	[1,1];	[1,1];	[1,1];	[1,1];	[1,1];	[1,1];	[1,1];	[1,1];	[0,0];
grp_proc_info.beapp_toggle_mods{'itpc',{'Module_On','Module_Export_On'}}	[0,0];	[0,0];	[0,0];	[0,0];	[0,0];	[0,0];	[0,0];	[0,0];	[1,1];
**FORMAT**									
grp_proc_info.src_format_typ	1;								2;
grp_proc_info.src_presentation_software									1;
grp_proc_info.src_data_type	1;								2;
grp_proc_info.src_linenoise	60;								60;
grp_proc_info.src_unique_nets	{'HydroCel GSN 128 1.0','Geodesic Sensor Net 64 2.0'};								{'HydroCel GSN 128 1.0', 'Geodesic Sensor Net 64 2.0'};
grp_proc_info.epoch_inds_to_process	[];								[];
grp_proc_info.src_eeg_vname	{'Category_1_Segment1','Category_1','Category1'};								
grp_proc_info.event_tag_offsets	0;								'input_table';[3]
grp_proc_info.behavioral_coding.events									
grp_proc_info.behavioral_coding.keys									
grp_proc_info.behavioral_coding.bad_value									
**PREP**									
grp_proc_info.beapp_toggle_mods{'prepp','Module_Xls_Out_On'}		1;							1;
**FILTER**									
grp_proc_info.beapp_filters{'Notch','Filt_On'}			1;	0;	0;	0;	0;	0;	0;
grp_proc_info.beapp_filters{'Lowpass','Filt_On'}			1;	1;	1;	1;	1;	1;	1;
grp_proc_info.beapp_filters{'Lowpass','Filt_Cutoff_Freq'}			80;	100;	100;	100;	100;	100;	100;
grp_proc_info.beapp_filters{'Highpass','Filt_On'}			1;	1;	1;	1;	1;	1;	1;
grp_proc_info.beapp_filters{'Highpass','Filt_Cutoff_Freq'}			4;	1;	1;	1;	1;	1;	1;
grp_proc_info.beapp_filters{'Cleanline','Filt_On'}			0;	0;	0;	0;	0;	0;	0;
**RESAMPLING**									
grp_proc_info.beapp_rsamp_srate				250;			250;	250;	250;
**INDEPENDENT COMPONENTS ANALYSIS**									
grp_proc_info.beapp_ica_type				2					
grp_proc_info.beapp_toggle_mods{'ica','Module_Xls_Out_On'}				1;					
grp_proc_info.happe_additional_chans_lbls{1}				[13,112];					
grp_proc_info.happe_additional_chans_lbls{2}				[9,58];					
**REREFERENCING**									
grp_proc_info.reref_typ					2;	4;			2;
grp_proc_info.beapp_reref_chan_inds									
**DETRENDING**									
grp_proc_info.dtrend_typ					1;	1;	1;	1;	1;

	Resting								Auditory
	Raw	PREP	Filter	HAPPE	CSD	REST	MskArt	RejPostSeg	ITPC
**SEGMENTING**									
grp_proc_info.beapp_toggle_mods{‘segment’,’Module_Xls_Out_On’}									
grp_proc_info.art_thresh				40;	3000;	100;	100;	100;	3000;
grp_proc_info.beapp_reject_segs_by_amplitude	0;	0;	0;	0;	0;	0;	0;	1;	1;
grp_proc_info.beapp_happe_segment_rejection	0;	0;	0;	1;	0;	0;	0;	0;	0;
grp_proc_info.segment_linear_detrend	0;	0;	0;	0;	0;	0;	0;	0;	0;
grp_proc_info.win_select_n_trials									
grp_proc_info.beapp_baseline_msk_artifact	0;	0;	0;	0;	1;	1;	1;	0;	0;
grp_proc_info.beapp_baseline_rej_perc_above_threshold									
grp_proc_info.win_size_in_secs	1;	1;	1;	1;	1;	1;	1;	1;	
grp_proc_info.beapp_event_code_onset_strs									{'stm+'};
grp_proc_info.beapp_event_eprime_values.condition_names									{'Standard', 'Native', 'Non-Native'};
grp_proc_info.beapp_event_eprime_values.event_codes(:,1)									[1,2,3];
grp_proc_info.beapp_event_eprime_values.event_codes(:,2)									[10,12,13];
grp_proc_info.beapp_event_eprime_values.event_codes(:,3)									[11,12,13];
grp_proc_info.evt_seg_win_start									-0.1;
grp_proc_info.evt_seg_win_end									0.8;
grp_proc_info.evt_analysis_win_start									
grp_proc_info.evt_analysis_win_end									
grp_proc_info.evt_trial_baseline_removal									
grp_proc_info.evt_trial_baseline_win_start									
grp_proc_info.evt_trial_baseline_win_end									
**POWER**									
grp_proc_info.bw(1,1:2)									
grp_proc_info.bw_name(1)									
grp_proc_info.bw(2,1:2)									
grp_proc_info.bw_name(2)									
grp_proc_info.bw(3,1:2)									
grp_proc_info.bw_name(3)									
grp_proc_info.bw(4,1:2)									
grp_proc_info.bw_name(4)									
grp_proc_info.bw(5,1:2)									
grp_proc_info.bw_name(5)									
grp_proc_info.bw_total_freqs									
grp_proc_info.psd_win_typ	1;								
grp_proc_info.psd_interp_typ	1;								
grp_proc_info.beapp_toggle_mods{'psd','Module_Xls_Out_On'}	1;								
**INTER TRIAL PHASE COHERENCE**									
grp_proc_info.beapp_itpc_params.win_size									0.256;
grp_proc_info.beapp_toggle_mods{'itpc','Module_Xls_Out_On'}									1;

Grayed out cells are where inputs can be left as default values, since relevant modules are not being run. See user inputs file in BEAPP, and user guide, for further information. [1]Users will need to set grp_proc_info.src_dir to the folder where their EEG files to be run are located. [2]Files were run in the order specified in the table from left to right, so that ‘format’ and ‘prepp’ modules did not need to be repeated for subsequent runs. [3]Because an offset table is specified for auditory data, offsets must be specified in beapp_file_info_table.mat.

Information table for baseline (.mat) files:

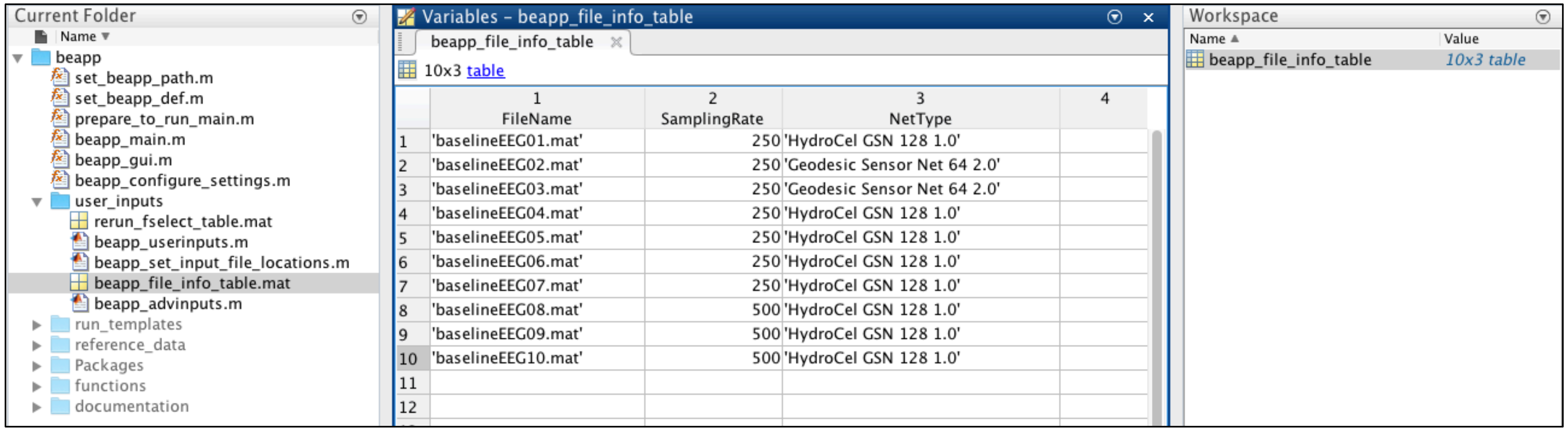

	1 FileName	2 SamplingRate	3 NetType	4
1	'baselineEEG01.mat'	250	'HydroCel GSN 128 1.0'	
2	'baselineEEG02.mat'	250	'Geodesic Sensor Net 64 2.0'	
3	'baselineEEG03.mat'	250	'Geodesic Sensor Net 64 2.0'	
4	'baselineEEG04.mat'	250	'HydroCel GSN 128 1.0'	
5	'baselineEEG05.mat'	250	'HydroCel GSN 128 1.0'	
6	'baselineEEG06.mat'	250	'HydroCel GSN 128 1.0'	
7	'baselineEEG07.mat'	250	'HydroCel GSN 128 1.0'	
8	'baselineEEG08.mat'	500	'HydroCel GSN 128 1.0'	
9	'baselineEEG09.mat'	500	'HydroCel GSN 128 1.0'	
10	'baselineEEG10.mat'	500	'HydroCel GSN 128 1.0'	
11				
12				

Information table for auditory (.mff) files:

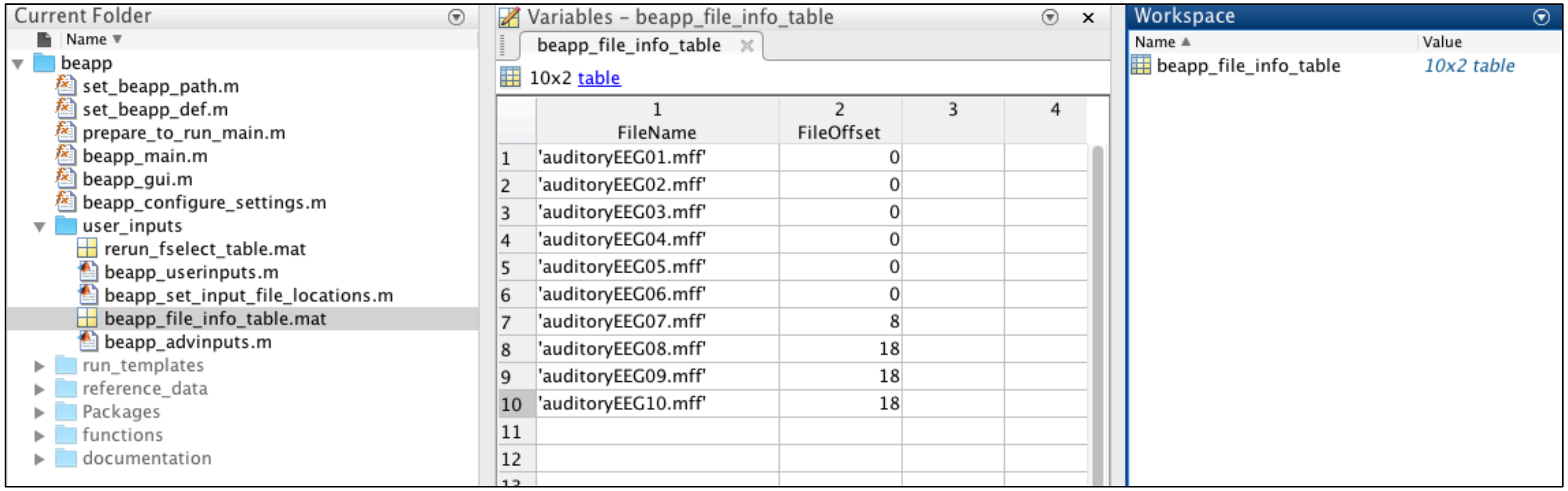

	1 FileName	2 FileOffset	3	4
1	'auditoryEEG01.mff'	0		
2	'auditoryEEG02.mff'	0		
3	'auditoryEEG03.mff'	0		
4	'auditoryEEG04.mff'	0		
5	'auditoryEEG05.mff'	0		
6	'auditoryEEG06.mff'	0		
7	'auditoryEEG07.mff'	8		
8	'auditoryEEG08.mff'	18		
9	'auditoryEEG09.mff'	18		
10	'auditoryEEG10.mff'	18		
11				
12				
